# Editorial Department

**Published:** 1856-02

**Authors:** 


Dr. E. R. Maxson, of Geneva, delivered the course of lectures on Theory and Practice of Medicine to the class of Geneva Medical College at its recent session. Prof. Sweetzer, who held the chair, resigned just before the commencement of term.

